# Integrating image caption information into biomedical document classification in support of biocuration

**DOI:** 10.1093/database/baaa024

**Published:** 2020-04-15

**Authors:** Xiangying Jiang, Pengyuan Li, James Kadin, Judith A Blake, Martin Ringwald, Hagit Shatkay

**Affiliations:** 1 The Computational Biomedicine and Machine Learning Lab, Department of Computer & Information Sciences, University of Delaware, 18 Amstel Ave, Newark, DE 19716, USA; 2 The Jackson Laboratory, 600 Main Street, Bar Harbor, ME, USA

## Abstract

Gathering information from the scientific literature is essential for biomedical research, as much knowledge is conveyed through publications. However, the large and rapidly increasing publication rate makes it impractical for researchers to quickly identify all and only those documents related to their interest. As such, automated biomedical document classification attracts much interest. Such classification is critical in the curation of biological databases, because biocurators must scan through a vast number of articles to identify pertinent information within documents most relevant to the database. This is a slow, labor-intensive process that can benefit from effective automation.

We present a document classification scheme aiming to identify papers containing information relevant to a specific topic, among a large collection of articles, for supporting the biocuration classification task. Our framework is based on a meta-classification scheme we have introduced before; here we incorporate into it features gathered from figure captions, in addition to those obtained from titles and abstracts. We trained and tested our classifier over a large imbalanced dataset, originally curated by the Gene Expression Database (GXD). GXD collects all the gene expression information in the Mouse Genome Informatics (MGI) resource. As part of the MGI literature classification pipeline, GXD curators identify MGI-selected papers that are relevant for GXD. The dataset consists of ~60 000 documents (5469 labeled as *relevant*; 52 866 as *irrelevant*), gathered throughout 2012–2016, in which each document is represented by the text of its title, abstract and figure captions. Our classifier attains precision 0.698, recall 0.784, *f*-measure 0.738 and Matthews correlation coefficient 0.711, demonstrating that the proposed framework effectively addresses the high imbalance in the GXD classification task. Moreover, our classifier’s performance is significantly improved by utilizing information from image captions compared to using titles and abstracts alone; this observation clearly demonstrates that image captions provide substantial information for supporting biomedical document classification and curation.

**Database URL**:

## Introduction

Knowledge-based biodatabases, e.g. Mouse Genome Informatics (MGI) (19) and WormBase (31), provide highly precise and well-organized information for supporting biomedical research. Much information in such biodatabases is manually collected and curated from the literature by domain experts. Typically, curators first need to scan through a vast number of publications to identify those articles that are most relevant to the database—in a process known as *triage*. Once the publications are selected based on assessing the full text of the documents, curators annotate detailed information within the text. However, the large and rapidly increasing volume of published articles and the limited ability of curators to quickly find all the relevant documents related to their topic of interest create a bottleneck in updating biodatabases with novel findings (3). One way to address this challenge is through automated document classification, that is, categorization of a large collection of articles by relevance to a specific topic. Such document classification can provide efficient and effective means to support the labor-intensive manual triage process. Hence, automated biomedical document classification is a topic of much recent research (1,5,15,20).

Much work aimed to address biomedical document classification over the past decade. The majority of the proposed methods focus only on information extracted from the title and abstract of the publications (1,6,8,9,11,12). For instance, Almeida *et al*. (1) employed various classifiers (i.e. Naïve Bayes, support vector machine) over a set of PubMed abstracts for addressing triage in the mycoCLAP database, which comprise articles discussing fungal proteins. Simon *et al.* (28) presented an online available tool, BioReader, that enables users to perform document classification using different algorithms over their own corpus.

However, as our group and several others have noted before (17,25,26), images convey essential information in biomedical publications. Accordingly, the image caption, which is a brief summary of the image, often contains significant and useful information for determining the topic discussed in publications. As such, several studies started incorporating information obtained from the captions for supporting document classification (5,13,14). Notably, most biomedical publications are stored in Portable Document Format (PDF), from which effective extraction of image captions is challenging. The PMC Author Manuscript Collection (24) provides a limited number of publications in plain text format, where figure captions are readily available for download.

In our own preliminary work (13), we presented an effective classification scheme for supporting triage in the Gene Expression Database (GXD), which aims to partition the set of publications examined by MGI into those that are relevant to GXD vs. those that are not. In that study, we trained and tested our classifier over a relatively small dataset of ~3000 documents, harvested from PMC, for which figure captions were available in addition to the titles and the abstracts. The proposed framework used a variety of features obtained from the different parts of the publication to assess the impact of using captions vs. title-and-abstracts only. Our classifier clearly showed improved performance by utilizing information from captions, titles and abstracts (0.876 *precision*, 0.829 *recall* and 0.852 *f-measure*) compared to using title and abstract alone (~0.770 precision, recall and *f*-measure). That early study has shown initial results, demonstrating the utility of image captions as an important evidence source for automatically determining the relevance of biomedical publications to a specific area. However, as noted above, the number of publications whose image captions are readily accessible (through PMC) is limited.

Another work using image captions as an aid for classification of a small dataset of ~1000 document, was reported by Burns *et al*. (5). They investigated the application of several word embedding methods including GloVe (22), FastText (4) and ELMo (23), combined with different neural network configurations for identifying scientific literature containing information about molecular interaction. The best performance attained over this small dataset was 0.820 *accuracy*. We note that the ‘black-box’ nature of neural-network classification systems makes the results hard to interpret, explain or justify, and as such—not as suitable for supporting a biocuration pipeline.

Notably, the above two classifiers were only applied to balanced datasets, in which all classes are of similar size. However, biomedical data sets are typically highly imbalanced, where relatively few publications within a large volume of literature are actually relevant to any specific topic of interest. Therefore, addressing class imbalance is essential for building practical classifiers in the context of biocuration triage.

Sampling strategies, which balance the number of instances in each class by either removing data from the majority class (*under-sampling*) or adding artificially generated data into the minority class (*over-sampling*), have been widely used in document classification in the face of data imbalance. In our preliminary work (12), we proposed a modified *meta-classification* scheme using a clustered-based under-sampling for addressing class imbalance. The presented under-sampling strategy employs *K*-means clustering to partition the majority class into subsets of documents, each covering a cohesive sub-area that has the potential to be individually distinguished from the minority class. Our reported performance over a set of ~90 000 PubMed abstracts with an imbalance ratio (i.e. the ratio between the number of minority to that of majority instances) of ~1:6, was 0.719 precision, 0.791 recall, 0.753 *f*-measure and 0.711 *Matthews correlation coefficient* (MCC) (18).

Given the importance of image captions for supporting triage, in this work we aim to integrate the information from captions into our preliminary classification framework described above, in order to develop an effective classifier that supports the triage task in GXD under class imbalance. Our reported performance over a large well-curated dataset consisting of ~60 000 PDF documents with a higher imbalance ratio of ~1:10 is 0.698 precision, 0.784 recall, 0.738 *f*-measure and 0.711 MCC. The experimental results demonstrate that by integrating information from captions, our classification scheme can effectively address an actual triage task in the face of data imbalance. Notably, our classifier is more efficiently applicable to biocuration triage compared to a previous proposed neural-network classifier (5) (see Section 3). Moreover, our statistical feature selection identifies significant terms that support the classification decision, thus making the class assignment easily interpretable.

## Materials and methods

### Data

In this work, we propose a binary document classifier using information gathered from the title, abstract and image captions to automatically identify the articles that are relevant to GXD within the MGI database. We train and test our classification framework over a large collection of publications curated by MGI throughout the years 2012–2016.

As the title and abstract parts of the scientific publications are readily available, we first download those from PubMed for each article examined by MGI for curation. As mentioned in the Introduction section, the full text of most publications is stored in PDF. To harvest image captions of *all* the publications curated by MGI that are available only in PDF, we applied PDFigCapX (16), a tool developed by our group, to extract images and their corresponding captions from the files. We thus constructed a dataset comprising 58 362 documents, where each document consists of a title, an abstract and captions for all images within the respective publication. Among these documents, 5496 are labeled as relevant to GXD comprising the *relevant set*, while the remaining 52 866 are labeled as irrelevant, comprising the *irrelevant set*. The imbalance ratio, as noted earlier, is ~1:10.

### Classification scheme

To address triage in the face of a large, imbalanced dataset, we follow the approach described in our earlier work (12). The proposed classification framework employs a meta-classification scheme, which combines the results produced by multiple simple classifiers (referred to as *base-classifiers*) into a single classifier (referred to as *meta-classifier*) to assign the final class labels to documents.

To train the base-classifiers, we first partition the irrelevant set into subsets using under-sampling to reduce the gap between the number of relevant articles and that of irrelevant ones. As we noted, the large irrelevant set covers a variety of distinct sub-areas (e.g. mutant alleles and phenotypes, Gene Ontology, tumor biology and quantitative trait loci), where each sub-area forms its own cohesive subset. Therefore, each such subset has the potential to be distinct from the set of relevant documents. Hence, we employ a cluster-based under-sampling strategy over the irrelevant set to split the irrelevant class into heterogeneous subsets, rather than the widely used random under-sampling strategy. Notably, as the motivation for the under-sampling step is to balance the size of each irrelevant subset with that of the relevant class, the number of clusters is determined based on the imbalance ratio within the dataset. Cluster-based under-sampling partitions the irrelevant class into topically coherent clusters, where each cluster corresponds to a subset of irrelevant documents covering a distinct topic; each such subset can be distinguished from the set of relevant documents by training an effective base-classifier.

**Figure 1:**
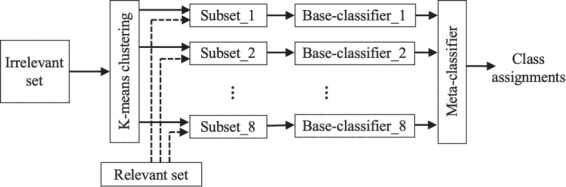
**Our classification framework.** The irrelevant class is partitioned into *k* (*k* = 8) subsets using *K* means clustering. Each such subset along with the relevant set are used to train eight base-classifiers. The results obtained from the base-classifiers are then used by the meta-classifier to assign the final class label to each document.

Specifically, we employ *K*-means clustering (2) to partition the irrelevant set into *k* clusters, using cosine distance as the similarity measure. As such, the large collection of irrelevant documents is divided into *k* subsets, each discussing a distinct sub-area. We then train *k* base-classifiers, each used to distinguish the relevant set from one of the *k* irrelevant subsets. As mentioned before, *k* is determined based on the imbalance ratio, which is ~1:10 within our complete dataset. We ran experiments modifying *k* in the range 8–10. Best performance was attained when *k* = 8, and thus we set the number of clusters *k* to 8 in the work reported here. We use Random Forest classifiers (10) as base-classifiers and support vector machines (SVMs) (7) for the meta-classification, as this configuration has proven effective in our earlier work (12). Figure 1 summarizes our classification framework.

### Document representation

*K*-means clustering is employed over the complete irrelevant set, while the base-classifiers aim to distinguish each of the irrelevant subsets from the same relevant set. We thus utilize different feature selection steps to represent documents when applying the *K*-means clustering from those employed for the base-classifier training/testing, as discussed below.

Our initial document representation is a variation of the bag-of-words model used in our earlier work, utilizing both unigrams and bigrams. Using a limited number of features to represent documents has proven effective in classification (27). To reduce the number of features, we first identify all *gene, enzyme, protein, mutation* and *anatomy* concepts using the publicly available biomedical annotation tools Pubtator (30) and BeCAS (21). We then substitute all gene and protein concepts by the generic term *PRGE*, while the specific mentions of enzyme, mutation and anatomy are similarly replaced by the generic term *ENZI*, *MUTN* and *ANAT*, respectively. We also remove standard stop words, single letters, rare terms and overly frequent terms (see details in (12)). The *Z*-score test, which we used in our earlier work, is then employed to select the features whose probability to occur within relevant documents *Pr*(*t*|*D*_rel_) is statistically significantly different from that within irrelevant articles *Pr*(*t*|*D*_irrl_). The higher the absolute value of the *Z*-score, the more significant the difference is between *Pr*(*t*|*D*_rel_) and *Pr*(*t*|*D*_irrl_). Thus, we consider a term *t* to be *distinguishing* with respect to our triage task only if the absolute value of its respective *Z*-score is >1.96 (which translates to $P<0.05$ (29)).

To employ *K*-means clustering over the irrelevant class, each document in the irrelevant training set is represented as an *m*-dimensional binary vector of the form ${V}^d=\Big\langle{V}_1^d,{V}_2^d,\dots, {V}_m^d\Big\rangle$, where *m* is the number of distinguishing features identified through the above feature selection, and ${V}_i^d=1$if the *i*th term appears in the document *d*, ${V}_i^d=0$ otherwise.

Notably, the above process is applied to the whole training set, selecting salient features for separating GXD-relevant documents from all other articles. As each base-classifier aims to distinguish between only one cohesive irrelevant subset and the relevant set, we employ the above feature selection strategy over each subset of documents used for training the corresponding base-classifier, with respect to the single set of relevant documents. Table 1 shows examples of distinguishing terms, selected through the above process, for each of the base-classifiers.

**Table 1:** Examples of distinguishing terms (right column) used for text representation when training/testing each of the eight base-classifiers (left column). The terms per classifier were obtained by applying our feature selection process over the corresponding sampled irrelevant training subset, with respect to the same *relevant* training set (recall that *ANAT* is the generic term used to replace anatomy concepts)

Base-classifier	Examples of distinguishing terms
1	average, change, follow, neuron, trace
2	ANAT weight, diet, fat, glucose, metabolism
3	antibody, inhibit, lysate, western, western blot
4	independent, immune, gate, vivo, cytometry
5	transcript, site, associate, sequence, fold
6	tumor, bind, cancer, inhibit, interact
7	wild type, stain, section, inflammatory, phosphoryl
8	scale bar, arrowhead, genotype, immunostaining, microscopy

The number of features selected (~19 000) for each base-classifier training/testing is still high given the number of documents in the training set (<8500 for each base-classifier, comprising the sampled irrelevant subset and the relevant set). To further reduce the number of features, we utilize *feature binning*, which we introduced in our earlier work, to group together those terms that share similar probabilities to occur in the relevant class, as well as in the irrelevant sets (12). Specifically, the continuous probability interval (0,1) is first divided into equal-width sub-intervals, each of width 0.0001, as such setting has yielded the best performance in our previous work (12). For binning a set of M terms, we start with M bins — each containing a single term. We iteratively merge two bins p and q into a single bin if and only if the probabilities of all terms *t_p_* in bin p and *t_q_* in bin q to occur within the relevant set, *Pr*(*t_p_|D*_rel_), *Pr*(*t_q_|D*_rel_), respectively) fall into a common sub-interval, while the respective probabilities of these terms to occur in irrelevant documents, (Pr(t_p|D_irrl), Pr(t_q|D_irrl)), also share a sub-interval.

When training/testing the base-classifiers, each document in either the corresponding sampled irrelevant training set, in the relevant training set or in the test set is represented as a binary vector ${G}^d=\Big\langle{G}_1^d,{G}_2^d,\dots, {G}_n^d\Big\rangle$, where *n* is the number of term-sets generated via feature binning, and ${G}_j^d=1$ if a term contained in the *j*th bin occurs in the document, 0 otherwise.

To train and test the meta-classifier, we apply the *k* base-classifiers over the complete dataset, where *k = 8* as discussed above. Each document *d* is then represented as an *8*-dimensional numerical vector *C^d^*=$\Big\langle{C}_1^d,{C}_2^d,\dots, {C}_8^d\Big\rangle$, where ${C}_l^d$ is the prediction score assigned by the *l*th base-classifier to document *d*, namely

\begin{equation*} {C}_l^d=\mathit{\Pr}\left(d\in\ \textrm{the relevant set}\ |\ {C}_l\right),\quad l\in \left\{1,\dots, 8\right\}, \end{equation*}

is the probability of document *d* to be identified as relevant by the *l*th base-classifier.

## Experiments and results

### Experiments

We conducted two groups of experiments, one for assessing whether the image captions indeed provide substantial information supporting the biocuration triage task and the other for comparing the performance of our classifier to that of a convolutional neural network (CNN) triage system presented by Burns *et al.* (5). To ensure the stability of the results, we employed stratified 5-fold cross validation in all experiments. In each cross validation run, 80% of the complete dataset is used for training and the remaining 20% is used for testing. When developing our classification scheme, the training set is further split, such that 75% of the training documents (60% of the original data) are used to train the base-classifiers while the remaining 25% (20% of the original data) are used for meta-classification training. All experiments were conducted on a Mac machine with 3.2 GHz 8-Core Intel Xeon W processor and Radeon Pro Vega 56 GPU.

To assess the impact of using image captions vs. title-and-abstracts alone, we first conducted three sets of experiments using the same meta-classification scheme while representing documents based on information extracted from the two different parts of the publications. In the first, we represented documents by applying our feature selection only to the title-and-abstracts of the articles. The average length of the title-and-abstract per document is ~200 words. In the second, we used only the information extracted from the image captions in our representation. The total image captions per article comprise ~1000 words on average. In the third, the title, abstract and all captions of each publication are concatenated and used as the information source for feature extraction and document representation.

As mentioned in the Introduction, in recent years’ neural network classification models have been proposed for supporting triage in biodatabases. We therefore compare here the performance of our whole system to that of an earlier proposed CNN classification framework (5). To employ such deep learning-based classifier, we first trained a 100-dimensional word embedding model using FastText over the training set, as such embedding configuration was reported to obtain the best performance (5). We then applied their classification scheme and compared the performance of such neural-network classifier with that of ours over the large imbalanced dataset we use here.

**Figure 2:**
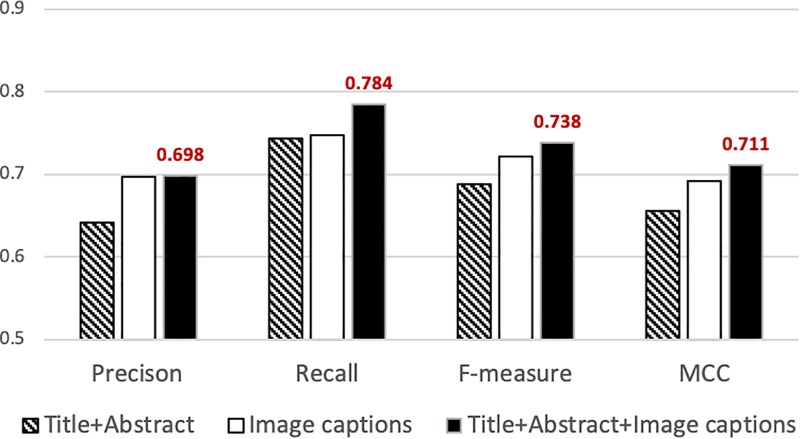
**Precision, recall, *f*-measure and MCC attained by our classifier using different sets of features.** The best performance is attained when articles are represented using features from titles, abstracts and captions (solid black bar). The highest values attained are indicated above the bar.

**Table 2:** Performance attained by our classification scheme compared to that attained using the CNN classifier proposed by Burns *et al.* (5). Standard deviation is shown in parentheses

Methods	Precision	Recall	*f*-measure	MCC
Our classifier	0.698 (.01)	0.784 (.02)	0.738 (.01)	0.711 (.01)
CNN classifier	0.740 (.04)	0.752 (.02)	0.745 (.01)	0.720 (.01)

### Results and analysis

We report the results using widely employed standard metrics, namely *precision, recall* and *f-measure*. We also report the MCC, which is a commonly used measure for evaluating classification in the face of data imbalance. MCC values range from −1 to +1, where −1 indicates total disagreement between the true labels and those assigned by the classifier while +1 implies total agreement; an MCC of 0 corresponds to random class assignment.

Figure 2 summarizes the results from the experiments in which we used information extracted from different parts of the publication for document representation. Our classifier attains the highest precision (0.698), recall (0.784), *f*-measure (0.738) and MCC (0.711) when we used features selected from the titles, abstracts and image captions (right solid black column in Figure 2). Such performance statistically significantly exceeds that attained based on the title-and-abstracts alone ($P\ll 0.001$, two-sample *t-*test for all measures). Furthermore, the precision, *f*-measure and MCC attained using image captions alone (0.696, 0.721 and 0.690, respectively) are statistically significantly higher than those attained using the title and abstract ($P\ll 0.01$, two-sample *t*-test). Such improvement strongly indicates that image captions indeed provide significant information for supporting biocuration triage. As described in the Experiments section, the average length of each title-and-abstract in our dataset is about 200 words per document, while the total image captions per document amount to about 1000 words on average. In the experiments where we use only the title and the abstract for training/testing the base-classifier, the number of features selected is ~5000, while the number of features identified when we use image captions alone is ~15 000. That is, as image captions comprise more words in total than titles and abstracts, captions provide a larger number of informative features for supporting classification.

Figure 2 also shows that combining title-and-abstracts with image captions attained significantly higher recall, *f*-measure and MCC ($P\ll 0.01$, two-sample *t-*test) compared to that of our classifier based only on image captions, while slightly improving the level of precision. The results demonstrate that our classifier effectively addresses triage under class imbalance by incorporating information from the titles, abstracts and image captions.

Table 2 compares the performance of our system attained when using information extracted from titles, abstracts and image captions to that of an earlier proposed CNN classifier (5). While the CNN classifier shows a higher precision, our classifier attains a higher recall indicating that more relevant documents have been identified. Moreover, while the CNN classifier shows slightly higher f-measure and MCC, the differences are not statistically significant as measured by the two sample *t*-test ($P>0.4$).

Notably, the statistical feature selection together with the classifiers we employ (Random Forest and SVMs) enables us to provide the significant terms supporting the classification decision (e.g. *gene manipulation, ectopic expression*) and thus provide an explanation/justification for the class assignments. Moreover, the distinguishing features we selected have the potential to support detailed document annotation as those features are important for identifying the topics discussed in the publications. In contrast, due to the inherent ‘black-box’ nature of neural networks, it is difficult to trace the decision-making process in the CNN classifiers. Moreover, timewise, each of the five cross validation runs of our classifier (see Experiments), has taken <40 min (wall clock), while each cross validation run of the CNN classifier has taken over 4 h (wall clock). As such, our framework takes less time to train than the CNN classifier proposed in (5), making it more efficiently applicable in the context of the biocuration triage task.

Last, comparing the implementation of our classifier with that of the CNN classifier reported by Burns *et al.* (5), we note that our classification scheme comprises several levels, including document representation and training of both the base-classifiers and the meta-classifier. The approach presented by Burns *et al.* adopts a pre-trained CNN configuration that uses available software packages and as such appears less complex. However, training such a neural network configuration requires setting over 10 parameters (e.g. the dimensionality of the word embedding vectors, the batch size and the number of epochs in the network training process, dropout rate). In contrast, our scheme requires setting only four parameters, namely the threshold defining rare terms, the threshold for defining overly frequent terms, the width of sub-intervals (*w*) for feature binning and the number of base-classifiers (*k*). Hence, our framework has fewer parameters and is simpler to set up compared to the neural network classifier.

## Conclusion and future work

We have developed a document meta-classification scheme employing features gleaned from titles, abstracts and image captions for supporting the biocuration triage task arising in GXD, over a large imbalanced dataset. Our classifier attains precision 0.698, recall 0.784, *f*-measure 0.738 and MCC 0.711. This level of performance demonstrates that our classification scheme effectively addresses imbalanced biomedical classification in GXD. The results show that using features obtained from the title, abstract and image captions of publications attains the highest performance, supporting the idea that image captions provide important and useful information for identifying the topics discussed within publications. Moreover, we show that our proposed classifier is as effective as a CNN classifier while being both more efficient and more transparent.

As image captions form an important information source for addressing triage and statistical feature selection can indeed identify meaningful terms supporting biomedical classification, we plan to develop an effective classification scheme using a variety of features obtained from image captions and associated sentences from the full-text discussing images, to further improve classification over large imbalanced datasets.

Ultimately, the tool described here will be incorporated into the GXD/MGI literature triage process. The selection of papers relevant for any areas of MGI corresponds to the first step of the MGI literature triage whereas the identification of papers for specific domains, such as gene expression, corresponds to the second step of the triage. The incorporation of the machine learning methods described here will support the selection of GXD-relevant papers and reduce the amount of manual curation required for this task.
